# 4-Meth­oxy-*N*′-(4-meth­oxy­benzyl­idene)benzohydrazide

**DOI:** 10.1107/S1600536811014012

**Published:** 2011-04-22

**Authors:** Ye Bi

**Affiliations:** a College of Chemistry and Chemical Engineering, Qiqihar University, Qiqihar 161006, People’s Republic of China

## Abstract

The title compound, C_16_H_16_N_2_O_3_, was prepared by the reaction of 4-meth­oxy­benzaldehyde with 4-meth­oxy­benzohydrazide in methanol. The dihedral angle between the two benzene rings is 3.1 (3)°. In the crystal, inter­molecular N—H⋯O hydrogen bonds link the mol­ecules into *C*(4) chains along the *b* axis.

## Related literature

For the biological activity of hydrazone compounds, see: Peng (2011 ▶); Angelusiu *et al.* (2010 ▶); Ajani *et al.* (2010 ▶); Horiuchi *et al.* (2009 ▶). For related structures, see: Zhang (2011 ▶); Lei & Fu (2011 ▶); Tang (2011 ▶).
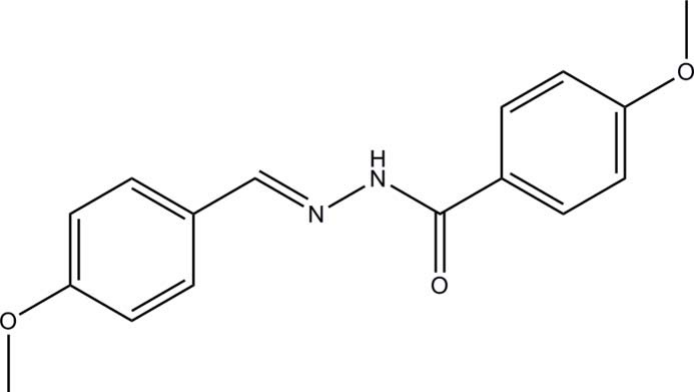


## Experimental

### 

#### Crystal data

C_16_H_16_N_2_O_3_
*M* *_r_* = 284.31
Monoclinic, 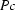

*a* = 10.617 (3) Å
*b* = 4.877 (2) Å
*c* = 13.632 (3) Å
β = 92.409 (2)°
*V* = 705.2 (4) Å^3^
*Z* = 2
Mo *K*α radiation
μ = 0.09 mm^−1^
*T* = 298 K
0.20 × 0.18 × 0.17 mm

#### Data collection

Bruker SMART 1000 CCD area-detector diffractometer
Absorption correction: multi-scan (*SADABS*; Sheldrick, 1996 ▶) *T*_min_ = 0.982, *T*_max_ = 0.984
3893 measured reflections
1396 independent reflections
1026 reflections with *I* > 2σ(*I*)
*R*_int_ = 0.029

#### Refinement

*R*[*F* ^2^ > 2σ(*F* ^2^)] = 0.059
*wR*(*F* ^2^) = 0.138
*S* = 1.30
1396 reflections
195 parameters
3 restraints
H atoms treated by a mixture of independent and constrained refinement
Δρ_max_ = 0.24 e Å^−3^
Δρ_min_ = −0.22 e Å^−3^

### 

Data collection: *SMART* (Bruker, 1998 ▶); cell refinement: *SAINT* (Bruker, 1998 ▶); data reduction: *SAINT*; program(s) used to solve structure: *SHELXS97* (Sheldrick, 2008 ▶); program(s) used to refine structure: *SHELXL97* (Sheldrick, 2008 ▶); molecular graphics: *SHELXTL* (Sheldrick, 2008 ▶); software used to prepare material for publication: *SHELXTL*.

## Supplementary Material

Crystal structure: contains datablocks global, I. DOI: 10.1107/S1600536811014012/cv5072sup1.cif

Structure factors: contains datablocks I. DOI: 10.1107/S1600536811014012/cv5072Isup2.hkl

Additional supplementary materials:  crystallographic information; 3D view; checkCIF report

## Figures and Tables

**Table 1:** Hydrogen-bond geometry (Å, °)

*D*—H⋯*A*	*D*—H	H⋯*A*	*D*⋯*A*	*D*—H⋯*A*
N2—H2*A*⋯O2^i^	0.90 (1)	1.99 (3)	2.844 (7)	157 (7)

Symmetry code: (i) 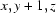.

**Fig. 1.**
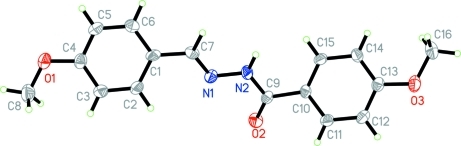
The molecular structure of (I) showing 30% probability displacement ellipsoids and the atom-numbering scheme.

**Fig. 2.**
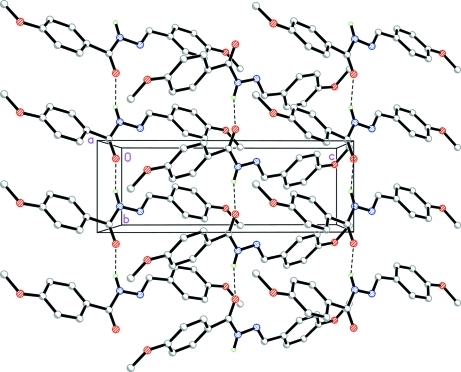
A portion of the crystal packing viewed approximately along the a axis. Hydrogen bonds are shown as dashed lines.

## supplementary crystallographic information

### Comment

Hydrazone compounds have attracted much attention due to their biological activities (Peng, 2011; Angelusiu *et al.*, 2010; Ajani *et al.*, 2010; Horiuchi *et al.*, 2009). In this paper, we present the title compound (I), which is a new hydrazone derivative.

In (I) (Fig. 1), all bond lengths and angles are normal and correspond to those observed in the related compounds (Zhang, 2011; Lei & Fu, 2011; Tang, 2011). The dihedral angle between the two benzene rings is 3.1 (3)°. In the crystal structure, intermolecular N—H···O hydrogen bonds (Table 1) link the molecules related by translation along axis *b* into chains (Fig. 2).

### Experimental

Equimolar quantities (1.0 mmol each) of 4-methoxybenzaldehyde and 4-methoxybenzohydrazide were mixed in methanol. The mixture was stirred at room temperature for half an hour to give a colorless solution. After keeping the solution in air for a few days, colorless block-shaped crystals were formed.

### Refinement

Atom H2A attached to N2 was located on a difference map and refined isotropically, with the N–H distance restrained to 0.90 (1) Å. Other H atoms were placed in calculated positions and constrained to ride on their parent atoms with C–H distances of 0.93-0.96 Å, and with U_iso_(H) set to 1.2U_eq_(C) and 1.5U_eq_(C8 and C16). In the absence of any significant anomalous scatterers in the molecule, 735 Friedel pairs were merged before the final refinement.

### Crystal data

**Table:**

C_16_H_16_N_2_O_3_	*F*(000) = 300
*M**_r_* = 284.31	*D*_x_ = 1.339 Mg m^−^^3^
Monoclinic, *P**c*	Mo *K*α radiation, λ = 0.71073 Å
*a* = 10.617 (3) Å	Cell parameters from 1579 reflections
*b* = 4.877 (2) Å	θ = 2.9–28.3°
*c* = 13.632 (3) Å	µ = 0.09 mm^−^^1^
β = 92.409 (2)°	*T* = 298 K
*V* = 705.2 (4) Å^3^	Block, colourless
*Z* = 2	0.20 × 0.18 × 0.17 mm

### Data collection

**Table:**

Bruker SMART 1000 CCD area-detector diffractometer	1396 independent reflections
Radiation source: fine-focus sealed tube	1026 reflections with *I* > 2σ(*I*)
graphite	*R*_int_ = 0.029
ω scans	θ_max_ = 26.5°, θ_min_ = 3.0°
Absorption correction: multi-scan (*SADABS*; Sheldrick, 1996)	*h* = −13→11
*T*_min_ = 0.982, *T*_max_ = 0.984	*k* = −6→6
3893 measured reflections	*l* = −16→17

### Refinement

**Table:**

Refinement on *F*^2^	Primary atom site location: structure-invariant direct methods
Least-squares matrix: full	Secondary atom site location: difference Fourier map
*R*[*F*^2^ > 2σ(*F*^2^)] = 0.059	Hydrogen site location: inferred from neighbouring sites
*wR*(*F*^2^) = 0.138	H atoms treated by a mixture of independent and constrained refinement
*S* = 1.30	*w* = 1/[σ^2^(*F*_o_^2^) + (0.0182*P*)^2^ + 0.6212*P*] where *P* = (*F*_o_^2^ + 2*F*_c_^2^)/3
1396 reflections	(Δ/σ)_max_ = 0.001
195 parameters	Δρ_max_ = 0.24 e Å^−^^3^
3 restraints	Δρ_min_ = −0.22 e Å^−^^3^

### Special details

**Table:**

Geometry. All esds (except the esd in the dihedral angle between two l.s. planes) are estimated using the full covariance matrix. The cell esds are taken into account individually in the estimation of esds in distances, angles and torsion angles; correlations between esds in cell parameters are only used when they are defined by crystal symmetry. An approximate (isotropic) treatment of cell esds is used for estimating esds involving l.s. planes.
Refinement. Refinement of F^2^ against ALL reflections. The weighted R-factor wR and goodness of fit S are based on F^2^, conventional R-factors R are based on F, with F set to zero for negative F^2^. The threshold expression of F^2^ > 2sigma(F^2^) is used only for calculating R-factors(gt) etc. and is not relevant to the choice of reflections for refinement. R-factors based on F^2^ are statistically about twice as large as those based on F, and R- factors based on ALL data will be even larger.

### Fractional atomic coordinates and isotropic or equivalent isotropic displacement parameters (Å^2^)

**Table:**

	*x*	*y*	*z*	*U*_iso_*/*U*_eq_	
N1	0.4613 (6)	0.2010 (11)	0.6226 (5)	0.0425 (15)	
N2	0.5326 (7)	0.2629 (11)	0.5429 (5)	0.0450 (16)	
O1	0.1067 (5)	0.2188 (12)	0.9836 (4)	0.0622 (17)	
O2	0.5985 (5)	−0.1728 (8)	0.5324 (4)	0.0565 (13)	
O3	0.8991 (5)	0.3171 (14)	0.1869 (4)	0.0609 (16)	
C1	0.3252 (7)	0.3481 (14)	0.7438 (6)	0.0426 (19)	
C2	0.3588 (7)	0.1499 (17)	0.8117 (6)	0.051 (2)	
H2	0.4308	0.0462	0.8022	0.061*	
C3	0.2908 (7)	0.0991 (17)	0.8929 (6)	0.056 (2)	
H3	0.3165	−0.0343	0.9381	0.067*	
C4	0.1832 (8)	0.2503 (16)	0.9059 (6)	0.048 (2)	
C5	0.1462 (9)	0.4446 (17)	0.8393 (6)	0.063 (3)	
H5	0.0728	0.5442	0.8479	0.076*	
C6	0.2175 (9)	0.4938 (16)	0.7594 (7)	0.063 (3)	
H6	0.1920	0.6292	0.7148	0.075*	
C7	0.3991 (8)	0.3890 (13)	0.6570 (6)	0.046 (2)	
H7	0.3992	0.5602	0.6269	0.056*	
C8	0.1325 (10)	0.0017 (19)	1.0495 (7)	0.073 (3)	
H8A	0.1294	−0.1691	1.0144	0.110*	
H8B	0.0708	0.0002	1.0990	0.110*	
H8C	0.2150	0.0258	1.0799	0.110*	
C9	0.6006 (7)	0.0565 (13)	0.5037 (5)	0.0379 (17)	
C10	0.6766 (6)	0.1469 (14)	0.4199 (5)	0.0358 (16)	
C11	0.7875 (7)	0.0084 (14)	0.4050 (6)	0.046 (2)	
H11	0.8132	−0.1311	0.4478	0.055*	
C12	0.8599 (8)	0.0742 (16)	0.3281 (6)	0.051 (2)	
H12	0.9361	−0.0157	0.3203	0.061*	
C13	0.8211 (8)	0.2711 (15)	0.2624 (6)	0.0434 (18)	
C14	0.7112 (7)	0.4090 (14)	0.2749 (5)	0.046 (2)	
H14	0.6848	0.5440	0.2305	0.055*	
C15	0.6390 (7)	0.3463 (15)	0.3546 (5)	0.0427 (18)	
H15	0.5644	0.4409	0.3635	0.051*	
C16	0.8644 (10)	0.5240 (18)	0.1176 (6)	0.062 (3)	
H16A	0.7837	0.4805	0.0870	0.093*	
H16B	0.9263	0.5338	0.0684	0.093*	
H16C	0.8596	0.6976	0.1504	0.093*	
H2A	0.539 (8)	0.440 (5)	0.525 (6)	0.080*	

### Atomic displacement parameters (Å^2^)

**Table:**

	*U*^11^	*U*^22^	*U*^33^	*U*^12^	*U*^13^	*U*^23^
N1	0.047 (4)	0.024 (3)	0.058 (4)	−0.002 (3)	0.015 (3)	−0.002 (3)
N2	0.045 (4)	0.040 (3)	0.052 (4)	−0.006 (3)	0.017 (3)	0.010 (3)
O1	0.055 (4)	0.071 (4)	0.062 (4)	0.008 (3)	0.025 (3)	0.005 (3)
O2	0.077 (3)	0.018 (2)	0.077 (3)	−0.002 (2)	0.027 (3)	0.007 (2)
O3	0.049 (4)	0.081 (4)	0.054 (3)	−0.001 (3)	0.016 (3)	0.008 (3)
C1	0.049 (5)	0.019 (3)	0.060 (5)	0.006 (3)	0.006 (4)	−0.002 (3)
C2	0.040 (5)	0.053 (5)	0.061 (5)	0.009 (4)	0.014 (4)	0.008 (4)
C3	0.043 (5)	0.066 (5)	0.060 (6)	0.012 (5)	0.005 (5)	0.013 (4)
C4	0.037 (4)	0.061 (5)	0.047 (5)	−0.004 (4)	0.006 (4)	−0.004 (4)
C5	0.056 (6)	0.063 (6)	0.071 (7)	0.026 (5)	0.017 (5)	0.009 (5)
C6	0.080 (7)	0.047 (5)	0.063 (6)	0.017 (5)	0.019 (6)	0.017 (4)
C7	0.061 (5)	0.016 (3)	0.063 (5)	−0.003 (3)	0.014 (4)	0.012 (3)
C8	0.085 (8)	0.072 (6)	0.066 (7)	−0.002 (5)	0.030 (6)	0.004 (5)
C9	0.031 (4)	0.038 (4)	0.045 (4)	−0.004 (3)	0.004 (3)	−0.007 (3)
C10	0.031 (4)	0.034 (3)	0.043 (4)	−0.010 (3)	0.006 (3)	0.000 (3)
C11	0.040 (5)	0.039 (4)	0.060 (6)	0.004 (3)	0.006 (4)	0.007 (4)
C12	0.038 (5)	0.058 (5)	0.057 (6)	0.010 (4)	0.009 (4)	−0.002 (4)
C13	0.039 (4)	0.042 (4)	0.050 (5)	−0.007 (3)	0.007 (4)	−0.007 (3)
C14	0.054 (5)	0.042 (4)	0.043 (5)	0.001 (4)	0.012 (4)	0.007 (3)
C15	0.037 (4)	0.038 (4)	0.054 (5)	0.007 (4)	0.005 (4)	0.001 (4)
C16	0.072 (7)	0.067 (5)	0.049 (5)	−0.011 (5)	0.017 (5)	0.003 (4)

### Geometric parameters (Å, °)

**Table:**

N1—C7	1.234 (9)	C6—H6	0.9300
N1—N2	1.383 (6)	C7—H7	0.9300
N2—C9	1.361 (9)	C8—H8A	0.9600
N2—H2A	0.900 (11)	C8—H8B	0.9600
O1—C4	1.371 (10)	C8—H8C	0.9600
O1—C8	1.408 (10)	C9—C10	1.492 (9)
O2—C9	1.186 (7)	C10—C15	1.367 (9)
O3—C13	1.366 (10)	C10—C11	1.380 (10)
O3—C16	1.421 (10)	C11—C12	1.364 (10)
C1—C6	1.370 (11)	C11—H11	0.9300
C1—C2	1.375 (10)	C12—C13	1.365 (11)
C1—C7	1.462 (10)	C12—H12	0.9300
C2—C3	1.369 (11)	C13—C14	1.364 (11)
C2—H2	0.9300	C14—C15	1.390 (10)
C3—C4	1.377 (11)	C14—H14	0.9300
C3—H3	0.9300	C15—H15	0.9300
C4—C5	1.359 (11)	C16—H16A	0.9600
C5—C6	1.373 (11)	C16—H16B	0.9600
C5—H5	0.9300	C16—H16C	0.9600
			
C7—N1—N2	117.1 (6)	O1—C8—H8C	109.5
C9—N2—N1	117.6 (5)	H8A—C8—H8C	109.5
C9—N2—H2A	124 (5)	H8B—C8—H8C	109.5
N1—N2—H2A	118 (5)	O2—C9—N2	123.4 (7)
C4—O1—C8	118.1 (7)	O2—C9—C10	123.4 (7)
C13—O3—C16	118.0 (7)	N2—C9—C10	113.2 (6)
C6—C1—C2	117.1 (8)	C15—C10—C11	118.6 (7)
C6—C1—C7	122.3 (7)	C15—C10—C9	123.9 (6)
C2—C1—C7	120.5 (7)	C11—C10—C9	117.4 (7)
C3—C2—C1	122.7 (7)	C12—C11—C10	120.6 (7)
C3—C2—H2	118.7	C12—C11—H11	119.7
C1—C2—H2	118.7	C10—C11—H11	119.7
C2—C3—C4	118.4 (8)	C11—C12—C13	120.4 (8)
C2—C3—H3	120.8	C11—C12—H12	119.8
C4—C3—H3	120.8	C13—C12—H12	119.8
C5—C4—O1	115.5 (8)	C14—C13—C12	120.0 (8)
C5—C4—C3	120.4 (9)	C14—C13—O3	124.2 (7)
O1—C4—C3	124.1 (8)	C12—C13—O3	115.7 (8)
C4—C5—C6	119.9 (8)	C13—C14—C15	119.5 (7)
C4—C5—H5	120.1	C13—C14—H14	120.3
C6—C5—H5	120.1	C15—C14—H14	120.3
C1—C6—C5	121.6 (8)	C10—C15—C14	120.7 (7)
C1—C6—H6	119.2	C10—C15—H15	119.6
C5—C6—H6	119.2	C14—C15—H15	119.6
N1—C7—C1	121.4 (6)	O3—C16—H16A	109.5
N1—C7—H7	119.3	O3—C16—H16B	109.5
C1—C7—H7	119.3	H16A—C16—H16B	109.5
O1—C8—H8A	109.5	O3—C16—H16C	109.5
O1—C8—H8B	109.5	H16A—C16—H16C	109.5
H8A—C8—H8B	109.5	H16B—C16—H16C	109.5

### Hydrogen-bond geometry (Å, °)

**Table:**

*D*—H···*A*	*D*—H	H···*A*	*D*···*A*	*D*—H···*A*
N2—H2A···O2^i^	0.90 (1)	1.99 (3)	2.844 (7)	157 (7)

Symmetry codes: (i) *x*, *y*+1, *z*.
